# Scholarly reading (and writing) and the power of impact factors: a study of distributed cognition and intellectual habits

**DOI:** 10.3389/fpsyg.2023.1165700

**Published:** 2023-07-17

**Authors:** Terje Hillesund

**Affiliations:** Faculty of Social Sciences, University of Stavanger, Stavanger, Norway

**Keywords:** distributed cognition, embodied cognition, scholarly reading, scholarly writing and publishing, intellectual habits, impact factor (IF), text creation

## Abstract

Using observational interviews and introducing theories of embodied and distributed cognition, this study examines the scholarly reading and the intellectual habits of a group of social scientists. All participants were working at universities in task environments dominated by digital artifacts and technologies. The study found a strong connection between scholarly reading and the scholars' writing processes and a further coupling to their digital publishing activity. While examining the participants' print and online reading, it turned out that their reading was so tightly coupled to their writing that this entanglement had to be at the core of the analysis. In the study, scholarly reading and writing are analyzed as cognitive processes that extend beyond the brain and body and comprise cognitive artifacts of texts and their material bearers, such as printouts, digital displays, computers, and the Internet. In the process of creating text—or reading *and* writing—brains, bodies, and artifacts are considered to be dynamically coupled in a distributed cognitive process. Based on interviews with a sample of academics, the study analyses how their scholarly reading relates to the other elements in such an extended process and how they utilize the affordances of cognitive digital artifacts in their creative and intellectual endeavors.

## Introduction

### Starting point and the aim of the study

Scholarly reading has become fully embedded in a digital working environment. Many scholarly research processes have been digitized and based on networks, especially the Internet. Currently, publication processes are digital, research results are published electronically, and scholars search for papers in effective search engines. Journal articles and e-books are skimmed online and sometimes downloaded (Tenopir et al., 2019). Research data are also collected and analyzed digitally, and the writing of articles is done onscreen, and so is most of the reading (Ibid.).

In this process, the importance of printed material is in steep decline, but printouts and printed books have not disappeared altogether (Tenopir et al., 2019), nor has the use of pen and paper. In addition, digital academic texts are still dominated by earlier print genres and typographical formats that stretch centuries back in time (Hillesund, 2005). However, even if the cognitive ecology of scholars may have the appearance of a hybrid system (digital plus print), digital technologies are dominant.

The primary aim of the study is to examine and describe the current characteristics of the scholarly reading and intellectual habits within this new digitally dominant cognitive-cultural ecosystem. With a focus on their physical handling of text, a sample of academics from text-intensive social sciences is interviewed about their reading habits (which early on proved to be closely tied to their writing). Their answers are analyzed using perspectives from embodied and distributed cognition theories.

In order to place the theoretical background of the study, the following sections give a short introduction to embodied reading research and its relation to different strands of embodied cognition theories. The purpose of the review is to relate the current study to the diverse range of studies in embodied reading.

### Theoretical approaches to the study of embodied reading

Nearly three decades ago, Chartier (1995) wrote that new material features of digital technology would inevitably and imperatively require new relationships to the written word, new ways of reading, and new intellectual techniques. Within reading research, the digital transformation of text has set off a cascade of research on the effects of digital reading. A number of experimental research have compared reading comprehension on printed paper and digital screens, very often using students as respondents. Results from this research are varying. However, meta-studies (Delgado et al., 2018; Clinton, 2019) have found a slight advantage of paper over digital reading for the reading of informational, but not for narrative texts.

Increasingly, research on digital reading and the comparison with paper reading has been criticized for being too narrow and out of touch with embedded and situated reading, or real-life reading. Coiro (2020) claims that one-way causal thinking is underpinning most analyses in current digital reading research, and she argues for a multifaceted perspective in the study of digital reading.

Along the same lines, Clowes (2019, p. 265) calls “the idea that the technology and artifacts of the Internet has a unidirectional and irresistible effect on the human mind” *the impact theses*. Mentioning Greenfield (2015) and Wolf (2018) as examples, he claims that the impact thesis is one-sided and sees Internet technologies as unidirectional forces that have mainly destructive effects on human cognition and reading.

In order to get a better grip on how the making of the meaning of the text is enacted in real situations, researchers have pointed to theories of embodied cognition, and they have coined the term *embodied reading*. Some have also argued for widening the range of methods to include phenomenological and ethnographical methods in research on embodied digital reading (Trasmundi et al., 2021; Hillesund et al., 2022). Theories of embodied cognition consider the role of the body and emotions in cognition and how cognition is dependent on humans' active engagement with the environment and with others (Varela et al., 1991). Within reading studies, researchers inspired by embodied cognition theories examine a whole range of areas and elements, such as neural activities in the brain, uses of the body, sensorimotor action, text materiality, reading technologies, and more recently, the reciprocal interaction between the elements in distributed reading systems. Some researchers have called this new focus *an embodied turn* in the study of reading (Trasmundi et al., 2021; Hillesund et al., 2022).

The overarching theory of embodied cognition can be divided into several fields of study and comprises of theories of *embodied, embedded, enacted*, and *extended* cognition (often called the 4Es), which all differ in certain ways. What this variety of outlooks have in common is an effort to overcome the Cartesian mind-matter dualism and a reaction to cognitivism and to computational and representational theories of cognition. These brain-bound theories of cognition are said to uphold the problematic dichotomies of mind–matter, brain–body, and an inner–outer divide (Varela et al., 1991; Lakoff and Johnson, 1999; Malafouris, 2013; Di Paolo et al., 2017).

In order to place different strands of embodied reading research (and this study) within the variety of embodiment theories, a rude classification of embodied cognition theories may be useful. In what is sometimes called a conservative (Rowlands, 2010) or weak (Gallagher, 2017) variant of embodied cognition, researchers are concerned with the relation between the brain and our experiences. According to Gallese and Lakoff (2005), the same neural and cognitive mechanisms that allow us to perceive and move around in the world also create our conceptual systems. Research within this strand has played a pivotal role in pointing to the importance of body and environment in human cognition, and it has influenced reading research. Empirical studies show that neural circuits that form functional clusters for *grasping* are active not only when we physically grasp but also when we hear and understand, and when we *read* sentences involving the concept of *grasping* (ibid.). Many experiments have shown that sensorimotor responses in the brain are elicited when participants read phrases relating to actions (Pulvermüller, 2005; Aziz-Zadeh and Damasio, 2008). Comprehension of narratives is also said to rely on mental re-enactments of perceptual and sensorimotor experiences (Engelen et al., 2011), and reading of a text is claimed to be accompanied by neural activity in the brain that *simulates* activities occurring during concrete interactions humans have with the environment (Glenberg et al., 2007). Despite a focus on body, action, and environment, within this line of thought, cognition proper is regarded as being located in the brain.

Another strand within conservative embodied cognition claims that bodily movements and actions themselves are pivotal for human perception and cognition; cognition is brought forth or enacted through our active engagement with and coupling to the environment (Varela et al., 1991; O'Regan and Noë, 2001; Noë, 2004). Based on these ideas of enactive cognition, researchers in embodied reading examine reading as a sensorimotor activity, as something we *do*, using our body, including our brain (McLaughlin, 2015). In this view, our bodies, especially our vision, touch, and our eyes, hands, and fingers, play active roles in perceiving text and in manipulating reading devices, and thus constitute a necessary part of reading, contributing to the making of the meaning of the text. According to this view, the act of reading varies with reading devices, and, using the concept of *affordances* (Gibson, 1995), researchers have examined how different reading technologies allow for different ways of holding, handling, perceiving, and interpreting the text (Hillesund, 2010; Mangen et al., 2019). Even if these studies are preoccupied with the significance of bodily movements and text materiality for reading, they often take for granted that technologies play a one-way causal role and examine how variations in materiality influence the meaning-making of reading, which ultimately is perceived as going on inside the body (including the brain).

As a critical development of the conservative model, theorists within a radical embodied position take as a basis a multidirectional and reciprocal view of causation. Inspired by dynamical systems theory (Kieverstein, 2018), they conceive cognition as a *distributed process* and claim that the elements in an organism's *dynamic coupling* with the environment are *constituting* human cognition (Rowlands, 2010; Newen et al., 2018). According to Rowlands (2010), proponents of radical extended cognition theory claim that cognition not only goes on in the head or in the active body, but that artifacts and the environment *co-constitute* the cognitive process. In the present study, the understanding of reading has been inspired by this latter line of thought, which will be presented in the following sections.

### Distributed cognition, distributed reading, and digital materiality

Proponents of radical embodied cognition theory claim that cognition is *extended*, an idea formulated by Clark and Chalmers (1998). They describe an *active externalism*, claiming that relevant external features are *active* and “because they are coupled with the human organism, they have a direct impact on the organism and its behavior” (p. 9). They state that “the relevant parts of the world are *in the loop*, not dangling at the other end of a long causal chain” (p. 9). In the current study, the ideas of Clark and Chalmers (1998) will be projected to the understanding of *reading*, and with their words it will be claimed that the external features (such as texts and devices) in a coupled reading system are ineliminable and just as causally relevant for reading as typical features of the brain. For reading, this means that changes in the properties of text technologies and text materiality will interact with changes in readers' behavior and thus change the overall process of reading.

In their article, Clark and Chalmers several times refer to Hutchins (1995), who in his book *Cognition in the Wild*, by ethnographically studying navy ship navigation, developed his theory of *distributed cognition*, a perspective that has influenced language studies (Cowley, 2011; Steffensen, 2015) and recently also reading research (Trasmundi et al., 2021). According to Hutchins (1995, 2014, 2020), cognition is not only an achievement of the individual brain, but rather distributed between persons and artifacts, between people in groups, and along different timescales, both short, within ongoing activities, and long, related to developmental and evolutionary timescales.

As for the cultural evolutional timescale, according to Malafouris' *material engagement theory* (2013), much of human cognition has evolved from our manipulation of objects and tools. In his book *How Things Shape the Mind* (2013), he makes an analysis of how our conception of exact numbers, of counting, and of quantity gradually grew out of physical manipulation of small clay objects used in trade and storage accounts in ancient Sumer. Eventually, numbers, numerical measurements, and calculations came to permeate most of modern life's thinking and actions, from time organizing, to speed limits, working hours, food prices, and instruments used to measure length, time, weight, and temperature. Based on material engagements, humans have historically developed a long line of cognitive tools, such as numbers, writing (also a Sumerian invention), charts, chronometers, calculators, and computers, tools that not only *ease* but also *are parts of* the cognising when we perform different tasks. Without these tools, much of our modern thinking and cognition would not have been possible (Hutchins, 1995).

According to Hutchins (2014), all humans grow into a culture of objects and cognitive artifacts; artifacts that at a basic level mediate our experience of the world. All of our cognition is thus situated, embedded, and inseparable from the cultural–cognitive ecosystem in which we live. This cognitive ecosystem, in addition to *things* (Malafouris, 2013) and *cognitive artifacts* (Hutchins, 1995), includes *social practices* and *institutional procedures* that describe how to achieve certain cognitive goals, such as how to do research and how to write an academic text (Gallagher, 2020). In these cases, much of the cognising is built into the institutional procedures, and many procedures are made possible by laboratory equipment and by modern computer systems. Even if cultural systems of reading, writing, and text along with numbers and calculating are often mentioned as forming the most influential of all modern cognitive ecosystems (Hutchins, 2014), a distributed cognition perspective on reading has only recently been explicitly formulated (Trasmundi et al., 2021).

Clowes (2019, p.260) claims that “while the situated/embodied perspective on human cognition holds that the environment needs to be central to our analysis of cognition, this seldom stretches to any serious analysis of the cognitive properties of artifacts”. However, research *has* been done on the cognitive properties of artifacts, such as Hutchins' study of tools in ships navigation (1995) and in airline cockpits (Hutchins and Klausen, 2012). Following (Hollan et al., 2000) proposal to apply distributed cognition as an integrated framework in human–computer interaction research, the cognitive properties and affordances of tools have been studied and efforts have been done to describe and conceptualize the roles of tools in cognition (Dix et al., 2004; Blandford and Furniss, 2006; Susi, 2006).

Nevertheless, in the evolving situation of digitisation and reliance on the Internet and cloud technologies, Clowes (2019) recommends applying theories that “attempts to offer a more satisfying version of the human relationship with technology” (p. 261), and he explicitly mentions the theories of Hutchins (1995, 2014, 2020), Clark and Chalmers (1998), and Malafouris' material engagement theory (2013). According to Clowes (2019, p. 261), material engagement theory “proposes that the human capacity for agency arises from the interaction of brains, bodies, and tools and puts the material stuff of artifactual culture center stage”.

As an illustration of his theory, Malafouris's (2013) most famous analysis is of the potter at the wheel, which beautifully explains the dynamic exchange between matter, body, and mind (Poulsgaard, 2020). As Malafouris observed in his studies, the potter does not simply create a pot from an initial mental image; rather, his hands and fingers feel and follow the clay as the pot *emerges* through dynamic transactions at the wheel. The same goes for the potter's mind; as the clay pot is emerging and undergoing continuous change, so are the potter's ideas of the pot. Thus, the creative agency is not resting with the potter alone but emerges in the dynamic coupling between the potter, wheel, and clay (Malafouris, 2013; Malafouris and Poulsgaard, 2020).

Following this line of thought, neither *social scientists* can be said to create a *scholarly tex*t from an initial mental image; they rather utilize affordances of digital technologies, and in the dynamical coupling of researcher and cognitive artifacts, the scholarly texts *emerge* and continually *change*, and so do the scholars' ideas of the texts. Of important cognitive artifacts in scholars' creative activities are other printed academic texts, which they study, reference, and cite. Thus, among scholars, reading is often an integrated part of a larger task, such as peer reviewing and writing articles or research proposals.

Having studied architects and using the material engagement perspective on their creative use of computers and design apps, Poulsgaard (2020) argues that there is a specific materiality to digital tools as well as to the potter's clay, a materiality he calls *digital materiality*. Poulsgaard (2020) says that digital materiality is constituted by the way digital tools operate and are configured and that the concept “describes the way successive layers of mathematics, code, and software come to mediate and shape architects' screen-based creative work” (p. 13). By including the workings of cloud software (Clowes, 2019) in the definition of digital materiality, much the same as Poulsgaard says about architects may be said of *social scientists*: “Just as molecular materials (such as clay), come to transform action with material objects (such as pots), so digital materiality come to enable and transform creative practices with computers” (Poulsgaard, 2020, p. 96).

The current study uses perspectives from embodiment theories (*embodied, extended, distributed* cognition, and *material engagement theory*), including Hutchins' (2014) concept of *cognitive-cultural ecosystem*, Gallagher's (2020) idea of *institutional cognition*, and Poulsgaard's (2020) definition of *digital materiality*. Based on particulars from observational interviews, the article describes and analyses scholarly reading and the habits of academics working in an environment dominated by digital artifacts, examining *how* material features of digital technology require new relationships to the written word, new ways of reading, and new intellectual techniques (Chartier, 1995).

### The analysis

In the analyses that follow, results from a 2009 interview study (Hillesund, 2010) will sometimes be referred to, thus without the current research being a comparative study. The 2009 study described how a group of academic readers in the humanities and social sciences used the Web and computers for overview in a manner characterized by browsing and skimming; that is by discontinuous and fragmented reading. Concentrated reading was usually done on paper, especially reading of long-form texts, but the study suggested that academics were seldom reading a scholarly article or book from beginning to end, but rather in parts, using hands and fingers flicking back and forth, underlining, and annotating, and often relating the reading to their own writing.

The current study and the 2009 study are not directly commensurate, but similar enough to make some comparisons. The 2009 study had more participants who were working with a wider range of subjects and were of a wider range of ages than the present 2022 study. The current 2022 study has a more strategically chosen and smaller group of young and established social scientists, and this study applies perspectives from embodied and distributed cognition, which the 2009 study did not. Still focussing on reading, the study examines how changes in parts of the cultural–cognitive system of research and publishing, especially within digital technology, have reverberated through the system and how younger social scientists have adapted to this new cognitive system.

## Methodology

The method in this study is motivated by cognitive ethnography and the way it is used in distributed cognition studies to examine how cognition (in this case reading) emerges in the interaction between people and artifacts, between people, and through time within a sociotechnical system. The typical techniques are interviews and observation (Hutchins, 1995; Susi, 2006) and video recordings (Hollan et al., 2000; Trasmundi and Cowley, 2020). The phenomenological first-person experience of the researcher is also a valuable resource in much qualitative research (Polkinghorne, 1988), or as Hollan et al. (2000) put it:

If one is to talk to experts in a meaningful way about their interactions with structure in their task environments, one must know what that structure is and how it may be organized. One must also know the processes actors engage in and the resources they use to render their actions and experiences meaningful (p. 179–180).

Thus, inspired by ethnographic and phenomenological methods, a combination of semi-structured interviews and observation was chosen (Smith and Osborn, 2015).

A sample of six respondents was strategically chosen among social scientists and consisted of three men and three women in their thirties and first half of their forties (which is relatively young within social science). They were all doctorates, of different nationalities, and of the six participants, five had permanent positions at Norwegian universities, two as professors, three as associate professors, and one post doc. The reason for this purposive sampling (Bryman, 2016) was to interview established scholars from text-intensive fields (with a tradition for reading) and of age low enough to be sure their academic training and early career had occurred after electronic publishing, Internet, and digital applications for searching, reading, writing, and publishing had become ubiquitous tools within (Western) academic institutions, that is after the turn of the millennia.

Within academia, there are no completely idiosyncratic ways of reading literature (or writing and publishing). By seeking analytic generalizations (Yin, 2014), qualitative studies with few strategically chosen informants were therefore believed to bring forth trustworthy knowledge about scholarly reading (and writing) within a working milieu dominated by digital technologies. In this study, the authoring researcher sat down with the academics and researchers (the participants) in their working place(s) (offices) and observed and discussed their reading and uses of books, digital devices, and printouts (their handling of materials and technologies), in short: their real-life academic reading. The aim of the study and the use of interviews was to enquire into characteristic features of digital reading and combined reading (digital plus paper) and to shed light on the relationship between text technologies and reading behavior among the participants.

During the interviews, the main focus was on the physical and bodily aspects of reading and the participants' use of different text media, such as stationary computers, laptops, tablets, printouts, books, and notebooks. The interviewees were asked to illustrate and describe how they arranged their working space, how they searched for literature, and how they navigated articles and books. They were asked when they used digital devices and printed paper, respectively. The participants were further asked how they used their fingers and hands when reading, about their underlining and note-taking, about text searches, and their retrieval of text passages for later use.

All the interviews proceeded in an informal fashion, and during the conversations, the participants frequently illustrated how they went about when reading and working onscreen and on printouts. This procedure was important. With reference to Ihde (1990), reading can be said to be a most familiar activity, solidly packed and sedimented. Thus, readers are prone to a kind of bodily absence or disappearance that occurs in the senses and parts of the body when engaged in reading (Leder, 1990; Hillesund, 2010) However, when illustrating their readings habits, the interviewer was able to observe what the participants were doing, and he could ask the participants to turn their attention to their physical handling of texts, whether on screen or on paper. Together, they could start a reflection on what the participants were actually doing and why they were doing it. In this dialogical process (and in the following analysis), information gathered from participants was deepened and enriched by the first-person experience of the interviewer, thus constituting a two-stage interpretation process, or a double hermeneutic, which is the basis of interpretative phenomenological analyses (Smith and Osborn, 2015).

All the interviews were recorded digitally and lasted 60 min or more. In three cases, follow-up interviews of 40–60 min were conducted. Soon after the recordings, the digital audio files were transferred into an encrypted area using VeraCrypt (encryption software) and deleted from the digital voice recorder. Transcription and other work on digitally identifiable personal data were done within the encrypted area. Handwritten notes and reflections were locked down and safely kept; all in compliance with the national regulations for storing and handling personal information in a research project (to which it was reported) and according to the agreement with the participants. The project reports to Sikt (Norwegian Agency for Shared Services in Education and Research).

Shortly after the interviews, the author carefully listened to the recordings and took notes on themes, important passages, observational notes, and analytic reflections. Most of the categorizing and analyzing was done in a combined examination of written transcripts and notes, and through repeated listening to the discourses. During the interviews and in the analytical phase, several unanticipated issues appeared, and some of the interviews were therefore followed up in order to cast additional light on these specific issues.

All participants are anonymised in this presentation.

## Results and discussion

### Initial findings

One of the early findings in the study was that there existed a very tight coupling of scholarly reading to the participants' writing process and their publishing. In the group of informants, scholarly reading was highly instrumental, closely tied to their writing and to their eagerness to get published. Such an extreme instrumentality was not anticipated in the study. It was, however, expected that scholarly reading had become almost exclusively digital, but this was only partly the case. However, compared to the 2009 study, the reading of books, especially printed books, played a less important role for the current interviewees, and so did the reading of printouts. Even if the use of printouts does continue, the significance of printed text has diminished, and the importance of digital text technologies has increased accordingly.

### Reading of printouts

As mentioned above, the informants still read printouts, but to a lesser degree than the group of informants did in 2009. In 2022, only very important articles pivotal to their current task were printed out, underlined, and annotated. As an example, Paul, a very digital-minded scholar, did not read much printed material, but he instinctively reached for his pen when asked to illustrate how he read a printout. He and all the participants were marking the printouts they were reading, using different tools and techniques; some were mostly highlighting, others using pen or pencil, and Sue used them all, but for a variety of purposes. In addition, the informants used post-it tags and sometimes notebooks at the side.

The participants had individual ways of positioning their bodies when reading printouts; some were sitting at their desks and others found comfortable positions in a good chair or on their office couch. Quite a lot of printout reading was also done out of the office, especially at home. Ken said he liked to read in cafés. None of the participants actively used the office desk space as a cognitive resource (Kirsh, 1995) the way the 2009 group did: In 2009 several of the scholars used to pile up printout of varying importance and to spread out prints and book on their desk for easy access and comparison (Hillesund, 2010).

As in 2009, everyone in the 2022 sample had personalized marking and annotation styles when reading printouts: Sue, for instance, closely followed the text with her pen, ready to underline important words and passages. All participants used highlighters, pens, or pencils to accentuate parts of the text, often varying pressure on the pencil or color of the highlighter to mark degrees of significance. The margins on the pages were used for vertical lines, exclamation marks, question marks, or for making notes and comments. Lisa had a habit of encircling certain words.

From an embodied point of view, the participants made use of the affordances of printed paper and writing tools, especially the overwritability of printed paper and the writing tools' ability to set lasting marks on paper; some participants even utilized the ability of pencils to vary the thickness of the line and marks. By fetching a pen or a highlighter, the participants actively engaged their fingers and tools in their reading, and as a multisensory and sensorimotor experience, the reading changed. The hands and tools became parts of the cognitive system within which the reading process took place.

At the same time, as the participants marked or annotated, the flow of their reading changed. However, the participants had great difficulty in verbally describing what happened when underlining or highlighting; they were not sure if they underlined and followed the text with their eyes simultaneously, or if they stopped reading at a certain point and let their eyes go back to fix the text anew and read the text once more when underlining.

Uses of hands and tools also change the text itself. Just as typographical features, such as italics and underlining, add meaning to words and texts, so do marking and annotating the text of printouts. The participants' underlining and encircling of words changed the materiality of the text and thus the meaning of it; the underlining or encircling brought forth or enacted a new meaning to phrases or words. Through marking and annotating, the participants introduced a personal form of agency and creativity and thus brought more of themselves into the reading. From an embodied cognition point of view, the pen or pencil functions as an extension of the hand and fingers, an extension that influences the visual system and lets the participants touch the text and change it.

Much of this activity is done almost automatically, so it is no wonder the participant had problems talking about the physicality of their reading. Reading is a sensorimotor habit and to a great extent a subconscious activity, and how, in neural terms, signification and meaning emerge through the marking and annotating activity is little known. However, based on experience, one could assume that the marking and noting in some way enhances the readers' recall and understanding of the text, and that it helps to relate its content to the readers' background knowledge, and to the task at hand. It certainly makes it easier to retrieve points from the text at a later stage.

### Reading and writing and the creation of text

Because readers make use of tacit knowledge and their acquired sensorimotor schemes (Di Paolo et al., 2017; Gallagher, 2020), the corporal aspects of reading were difficult to describe verbally and even to illustrate authentically in the interview setting. However, the participants in the study were more loquacious when asked about the reasons for their printing out and annotating certain texts. They said it was because these texts were “important” or “central to their current task”, which usually was to design a research project or to write an article.

Martin was the only participant that said the primary reason for reading printouts was to improve his understanding of the text and to get a better grip on of the author's intentions and meaning with the text. He related underlining and notetaking—his bodily engagement with the text—to in-depth reading and to enhanced understanding of central theories and tenets of the field of study he was interested in—and to the research and the writing he was currently preoccupied with. Martin's answers to these questions were very much in line with the findings from the 2009 study in which comprehension and recall were the reported reasons for the then participants' marking and annotating.

In contrast to Martin, the rest of the 2022-participants clearly articulated an instrumental attitude toward their paper-based scholarly reading. The most important reason for their printing out and marking certain texts was these papers' relevance for their writing of papers; the underlining and marks were made to be able to recall and to reference theoretical point and empirical results in their own texts. The same reasons were given for their notes and marginal reflections. In addition to underlinings and annotations, Ken said he made a list on the first page of the printouts of what points they contained and where to find them. He said that for each article he wrote himself (or in collaboration with others), he used four to five printouts. “There,” he said, and fetched a small bundle of printouts, telling the interviewer that he kept them for now because the article he had written was still in review and that he could get use of them if he had to revise his article. A limited number of printouts for each project was common among the participants, and contrary to the 2009 participants, who were notoriously anxious to print out their own manuscripts for inspection and editing, the 2022 participants seldom took printouts during writing of their own articles.

As the discussion of reasons for reading evolved, all the participants told stories of how tightly intertwined their reading was with their writing and publishing. Ken said that he and a collaborator recently had collected data in the form of online interviews, and that they had had ideas for three different papers. When he had assessed, downloaded, and skimmed enough articles onscreen and also read some as printouts, he had got enough inspiration to start writing the introduction of the first article. He said that “after I have started writing, I continue reading, and then I continue writing”. The reading gave him new ideas and so did the writing, and he continued to go back and forth between his writing and his reading: “So, I'm writing, but I'm still reading at the same time”. However, even if the printouts were “core texts”, as he said, most of his reading was done onscreen as part of searches done in parallel with his writing.

Similar patterns of intertwining of writing and reading were common in the whole group. One of the participants characterized her reading as “extremely instrumental”. When she and her co-writers had decided to which journal they wanted to submit their manuscript, they searched previous numbers of the journal for articles and scholars to reference in their own paper. In order to position themselves, and thus increase their chances of acceptance by the reviewers and editors, they actively consulted topical articles for relevant points when writing. Another participant said that many of the authors and papers she referenced she had only skimmed and read in parts, and often she had only read the abstract. Yet another interviewee said that he simply skimmed most of the papers he had found relevant for his writing, searching for theoretical points and empirical findings, claiming that he only read scholarly content that was “publishable”, admitting that many of the papers he soon deleted and forgot.

This intertwining, or subsuming of reading under writing, seemed to give little space for reading of scholarly books, and Lisa's story is illustrative: When she needed to reference a book, she found an electronic version online, via the library system or Google Books, searched it and found the parts she needed for her writing and referencing. She never cited any literature that was not available online. The books the participants in this study most typically read in entirety were the curricula books they also expected their students to read.

In the present study, the participants' scholarly reading is described and analyzed as a cognitive process that extends beyond the brain and the body (Clark and Chalmers, 1998) and comprises of constitutive artifacts of written texts and of their material bearers, such as printouts, printed books, digital displays, and computers (including hardware, software, and paraphernalia), along with pens and pencils. According to radical embodiment theories, in the process of reading, brain, bodies, and artifacts are dynamically coupled, and the possibilities and constraints of the artifacts will influence the process. However, as the interviews illustrate, cognitive processes, such as writing, are not merely distributed across humans and artifacts; they are situated in a task environment and are often distributed across many people in cooperative activities (Hutchins, 1995), such as in co-writing, in seminar discussions, and during the process of publishing in which peer reviewers and editors, through their reading and comments, are co-creators of the finished article.

In addition, cognition, such as scholarly reading, always have a temporal distribution across different timescales (Hollan et al., 2000; Hutchins, 2014, 2020,). At a micro scale, the here-and-now of reading incorporates retention of what words have just now been perceived and anticipation of what words are about to come, and sometimes, the acts of underlining and highlighting. At a meso scale, the sequences of reading are parts of an ongoing activity or task, such as writing a paper, and at a macro scale the reading activities are enabled and constrained by institutional norms and cultural evolution, such as the historical development of writing, of script technologies, and of different scholarly genres.

To take all this into account in a single analysis is not possible (Hollan et al., 2000; Hutchins, 2014). However, to delimit a manageable analytic level of the scholarly reading process is neither an easy task. Such a delimitation may be less problematic for the process of reading a printed newspaper, which for analytical purposes may be regarded as a standalone activity. In the newspaper case, a limited study of the coupling between reader and artifact—that is between body (including brain) and newspaper (including text)—may be productive. However, as the interviews in this study have shown, scholarly reading is often tightly intertwined with scholarly writing, and even more so as computer systems allow for working methods in which the processes of reading and writing are easily combined into the larger process of creating a text. This activity, the creation of a text, is therefore a more relevant and productive level of analysis, even when the primary study is that of reading.

### Searching

In order to create an academic text, scholars usually have to search for appropriate literature to include and reference in their text. For the interviewees in this study, Google Scholar was the most important search engine, and much more used than the national search portal Oria, which includes most larger portals such as JSTOR, Science Direct, and Scopus. Search habits varied among the participants, and in addition to Google Scholar and Oria, relevant literature was found at scholarly social media sites (ResearchGate and Academia), in e-mail notifications from journals, and from recommendations given by colleges and co-writers. All participants also searched and found literature in reference lists, footnotes, and endnotes in papers and e-books they were reading. If no active link or DOI was available in these texts, the title or the author's name was cut and pasted into the search bar of Google Scholar, which functioned as a hub for many of the other methods of searching. Sarah, typically, said she could not imagine doing research without Google Scholar.

The reason for the popularity of Google Scholar was 3-fold: the ease of use, good results, and the easily assessable bibliometrics. When scrutinizing the results of searches, three elements decided whether a title caught the participants' interest: topic (if the title suggested relevance for their writing), impact (how many times the contribution had been cited), and the standing of the author. If the author was unknown to the participants, they often checked their bibliometrics in their profile page on Google Scholar (citation statistics and h-index), and Lisa said she did not like it when an author did not have such a page. On the profile page, Lisa said she used to check if the current author had other interesting, high-impact contributions related to their topic.

The preoccupation with impact factors also applied to scholarly journals. Everyone wanted to publish in high-ranked journals, and their searches and writing were partly aimed at convincing editors and peer reviewers, making them the immanent readers of their texts. When Sarah spoke of her searching habits, she simultaneously talked about her experiences with submitting papers to journals and of what she believed peer reviewers were looking for when they read a manuscript. While talking, Sarah showed the interviewer how portals, such as Taylor & Francis Online and Cambridge Core, presented the journals' overall impact factors and the individual articles' metrics. Generally, the interviewees had very good knowledge of impact factors and publication frequencies, and this knowledge guided their search activity.

Searching is in itself a reading activity, and when an article had caught the participants' attention, a new round of reading followed. Still online, the interviewees accessed and considered whether the opened article could be used in their writing, either in a literature overview or to support their current paper's arguments or theoretical discussions. Reading the abstract often sufficed, but sometimes the interviewees went on to skim the introduction, and maybe the conclusion. Martin and Sarah used to look at the empirical results, the statistics, to check for quality and relevance. At this stage, many papers were considered to be uninteresting, but once in a while, large parts of (and even whole) articles were read online.

When this search for relevant articles was done in the midst of a writing session, paraphrases of and references to the online articles were sometimes written directly into the manuscript, and the referenced article was then closed and left. More often, however, the documents were downloaded to the directory of the computer for current and later use. In this process, the participants had individual ways of sorting and storing relevant literature, usually setting up folders for each paper or folders to indicate importance or urgency, and often also using their personal system of re-naming the documents for easy retrieval. For the participants, this storage of relevant literature was indispensable for their write-read-write process of creating a scholarly article.

### Embodied text creation

For the participants, digitally creating a scholarly article—by searching, reading, and writing—required high levels of skills: relevant background knowledge within their field of study, experience with academic genres, knowing how to handle a multitude of hardware and software, and high levels of sensorimotor skills.

The latter pertains to the corporal aspect of digital work. For the interviewed participants, their bodies were active in reading and writing onscreen, with variations in their physical working habits, and in their ways of organizing the task environment; they differed in where and how they read, in how they sat, and if they used two large screens, such as Sarah, or a tiny laptop, such as Lisa. Common for the participants was that they no longer used the three-dimensional space of their office desk as a cognitive resource (Kirsh, 1995) in the way the participants were doing in the 2009 study. Most of their spatial manipulation had been transferred to the two-dimensional computer desktops, where the participants had developed slightly different, but skilful ways of navigating the many windows and software interfaces needed to read, write, and publish a text.

In the interview, Sue said she felt a distance between herself and the digital text caused by the indirect connection between mouse and screen and between keyboard and screen, a distance that she did not feel when reading printouts and using a pen or a pencil. Among researchers, similar experiences have made them claim there is a decreasing materiality from print reading to screen reading, and thus a decreased embodiment (Schilhab et al., 2018), but this notion may be misleading. Rather than a *decreasing* materiality there is a *difference* in materiality of printed and digital texts and their corresponding media.

In print, patterns of ink are applied to the surface of paper and these patterns both record the text and make it visible and legible in a fixed material form (Hillesund, 2005; Hillesund and Bélisle, 2014). This physicality makes the participants' reading of books and marking of printouts obviously embodied; they must use their hands, grasp, and hold a book in the focal area of the eyes when reading (McLaughlin, 2015). On a printout they must use their fingers and a pencil or a pen to be able to draw a line onto the printed paper.

In a digital environment, by contrast, storing and representation of text are done in separate operations, and the fixity of the text may seem to dematerialise. In internal computer systems, written texts are stored as binary digital digits. From storage systems, encoded texts are fetched by users and presented onscreen in a multitude of software applications, such as readers and browsers, and also in word processors, in which the scholars can edit, recompose, or expand the text. Digital texts are thus malleable and flexible and may be less physically tangible, but their reading and editing is no less embodied, and the text does have a materiality: To be read and manipulated a digital text must necessarily be presented to the participants in a legible form, usually by the use of pixels on a screen, and thus have a certain form of physical materiality (Hillesund and Bélisle, 2014).

Accordingly, screen use is fundamentally embodied; the participants in the study revealed high levels of physical skills when using a mouse or a touchpad and directing the cursor around the screen, clicking, doble-clicking, scrolling, copying, dragging, and dropping, intermittingly writing on the keyboard. McLaughlin (2015) claims that even the easiest task, such as opening and reading an e-mail, requires highly complex manual skills. Simply to move the cursor over the screen requires a finetuned eye-hand coordination in which “the eyes move in “ballistic saccades” to the desired target” (p. 169) and the hand on the mouse (or the finger on the touchpad) simultaneously moves the cursor to the target, being the target an icon, a tab, a search bar, or a position within a text.

Most of the participants' moving around the screen was done almost automatically. In embodiment terms, the participants were activating their sensorimotor schemes, which had been established through repeated action. However, the skills are not pertaining to physical aspects of computer use alone. Proficient movements are always already informed by “embodied knowledge of the options available in the system” (McLaughlin, 2015, p. 169), or, in the language of embodied cognition theory: of the learned affordances of hardware *and* software. Whether using two large screens or a small laptop screen, the participants in the study moved with ease between different windows and software applications, be it for searching, storing, reading, or writing. Through their onscreen and online actions, they showed an intimate knowledge of many of the affordances of the computer system and how to use the software in their screen-based creative activity. This creative activity comprised not only of their writing, but also of their use of digital tools for collecting, analyzing, and presenting data.

So, the *materiality of computer systems* both enable and constrain the embodied activity of scholars. As for *physical materiality*, buttons have to be pushed to start the machine, and the mouse or touchpad and movement of the curser operate in certain ways that everyone has to comply to if they want to use the computer. Furthermore, the onscreen graphic layout guides the eyes and the hand-and-curser movements of the users to the intended target. Similarly, *digital materiality* is constituted by the way operating systems and software applications operate and are configured. To be successful, the participants in the study had to follow the software procedures and scripts when they wanted to open programmes, find and manipulate documents, surf the Internet, or to read or write a text. Thus, both physical and digital materiality constrained their actions. However, within the combined physical and digital constraints, computers offer a wealth of possibilities and the participants in the study were well accustomed to “engage digital tools creatively to build upon the opportunities that different software provide” (Poulsgaard, 2020, p. 22).

### Distributed text creation

As described above, when the participants were creating a text, they were coupled to the computer system, including the Internet, in their searching, reading, and writing activities. However, the creative agency was not resting solely with the individual participant; it *emerged* in a dynamic coupling between participant, computer, and text (Malafouris, 2013; Malafouris and Poulsgaard, 2020; Poulsgaard, 2020). The computers and networks, with their hardware and software, were ineliminable parts of the cognitive process of creating, and later publishing, their articles. The features of the computer system can be said to have been co-constituting the cognitive activity in which the interviewees participated. During their scholarly work, the computers were not external to the participants; the computers were dynamically coupled to their bodies and brains and had an impact on their behavior (Clark and Chalmers, 1998). Generally, computers have what Malafouris calls *material agency* (2013), and the combined physical and digital materiality of computers have an impact along several timescales: on the participants' immediate sensorimotor acts, on the coordination of these acts according to task (the writing of articles), on the development of sensorimotor habits, and on mastery of the possibilities of the computer system, a mastery that ultimately interacts with changes to the structures and functions of the participants' brains (Anderson, 2014).

The interviewees' screen-based working habits demonstrated that scholars are not only dynamically coupled to the computer system but also to the Internet and its cloud technologies (such as artificial intelligence) (Clowes, 2019). The cognitive process of creating a text (reading *and* writing) extends into cyberspace and was distributed among the participants and their online collaborators (co-writers, colleagues, peer reviewers, and editors), cognitive artifacts (computers and the Internet), and institutional procedures, especially those of scholarly journals. This distribution of cognitive work was particularly evident in the participants' continuous efforts to get published. In this process, scholars have to conform to strict requirements of subscription schemas, genre norms, article lengths, and quality criteria of the desired journals. Even if it was not explicitly asked for in the interviews, the interviewees spoke at length about journals, peer reviewers, and editors when explaining the cognitive processes of reading and writing. They had extensive experience with submission patterns, document formats, peer review processes, publication routines, and impact factors, and this experience pervaded their reading and writing.

From a radical embodied cognition point of view, the participants' interactions with peer reviewers and editors make them (the reviewers and editors) co-constitutive of the cognitive process of writing. Thus, the process of creating articles was widely distributed within a cognitive system that, in addition to scholars, manuscripts and other digital artifacts, included “social processes and institutional procedures describing how to achieve certain cognitive goals” (Gallagher, 2020, p. 215). The historically established procedures of academic publishing are now to a large degree mediated by the net-based systems of electronic scholarly journals. The journals' digitized procedures, including the peer reviewing, play important roles in the cognitive process of creating articles. In this process, the scholars merely play a part, a part that is nevertheless pivotal in re-creating and upholding of the current publishing systems and the academic institutions, an observation that seems to affirm Hutchins (2011) claim that “reading and writing are cultural practices *par excellence”* (2011, p. 441).

### Continuous publishing

From the very beginning of a project, during the online search for relevant literature and relevant journals, different measures of impact were assessed by the participants and thus indicative of what they would eventually read. During the interviews, several participants expressed an ambivalent attitude toward this activity. They had cracked the publishing code, and from the point of view of the academic system, they had done well; they were doing research and publishing results. However, the interviewees felt a constant pressure to produce and claimed a high publication rate was necessary to get a permanent tenure, to get research funding, and to achieve a high standing within the academic community. Sue was discontent with the “extreme instrumentality” of academic reading, and Ken said that he, after all, did not do ground-breaking research when commenting on his write-read-write efficiency. Paul complained he did not have time to read anything outside of his writing, and Sarah said she probably would get a more meaningful academic life if she let go the impact pursuit and used more time on her main interests.

From the point of view of distributed cognition and digital materiality, it is interesting to observe that the academic turn of attention toward quantitative measures of quality (impact factors) is made possible by the digital materiality of text, computers, the Internet, and a variety of cloud technologies (Hillesund and Bélisle, 2014; Clowes, 2019). The cloud technologies make it possible to gather extreme amounts of data, make statistics and comparisons on everything from individual publication rates, journal impacts, and university standings. Listening to the interviewees' stories, a preoccupation with metrics and impact factors seems to have permeated the academic system *via* research authorities, university leaders, journals, and all the way into the bodies of the study's participants as their fingers decide the strength of pencil-pressure with which to underline a text in a printout.

## Conclusion

The initial aim of this project was to study scholarly reading among a group of social scientists. However, the interviews early indicated that the participants' reading was so tightly coupled to their scholarly writing that this entwinement had to be at the core of the analysis. The participants' creation of text—their combined writing *and* reading—was further found to be closely entangled with their publishing activities and partly driven by their efforts to get high bibliometric scores.

The article has examined the participants' embodied print-based and digital reading. Their creation of text was analyzed as a distributed cognitive process in which scholars, cognitive artifacts, social interactions, and institutional procedures are dynamically coupled, forming a cognitive system. The findings suggest that the participants do not create their scholarly articles from an initial mental conception, but rather that their scholarly articles emerge and change through a dynamical coupling with cognitive artifacts (mainly computers) and other academics. Simultaneously, their ideas of the texts change.

Interestingly, writing of the present article illustrates these findings. Starting with a focus on scholarly reading, the interviews and the efforts to formulate and discuss results soon brought forth new ideas and perspectives. As writing of the paper progressed, a growing realization of how crucial write-read-write processes were for the participants' reading and for their creation of text caused a need for reorientation, and new rounds of searching, reading, and writing followed, much of what was done in a coupling of author, computer(s), software, and the Internet. New ideas formed, and the current article changed into an examination of the interviewees' distributed process of creating scholarly texts, which ultimately lead to a better understanding of their scholarly reading.

The present article describes how a group of academics in their thirties and early forties utilize affordances of digital technologies and how repeated enactments of their skills establish new intellectual habits. Whether these descriptions and analyses are representative for academics in other geographical areas, fields of study, or age groups, is an interesting empirical question, but not the focus of this enquiry. It may, however, be a topic of further enquiries.

In addition, the use of observational interviews in this study has many limitations, and more research has to be done to better understand the process of reading within a digital task environment. As this study indicate, much scholarly reading is intertwined with writing at several levels of the writing process. At a basic level, reading is involved in the physical forming of word, sentences, and paragraphs. When the written text has materialized (in physical or digital form), a new process of re-reading and re-writing is often carried out. During the writing process, reading of other texts give ideas of new arguments to include via paraphrases, comments, references, and citations. The write-read-write process of creating text can thus be a highly creative way of thinking (Menary, 2007).

Within digital technology writing (including reading) and publishing processes have been streamlined and most of the activity is carried out onscreen. However, the current study gives few details on the concrete and complex embodied sensorimotor actions (and online interactions with others) that the participants carried out in this process. Other methods such as video recording, eye-tracking devices, applications for monitoring mouse (track-pad) and cursor movements, and brain-imaging (all in combination with interviews) will probably give interesting data on how scholars actually use their senses and bodies (mostly hands, fingers, and brains) when they read, search, save, copy, retrieve, move, rearrange, rewrite, and type text, and thus utilize the affordances of computers and Internet (artifacts) in the distributed cognitive process of creating a scholarly text, such as a journal article. Theoretical inspiration from other fields, such as human–computer interaction and neuroscience, could also be fruitful in order to describe, characterize, and explain the cognition enacted in the digital cultural–cognitive ecosystem that is about to dominate academia.

By applying embodied and distributed cognition theories on the study of reading, writing, and intellectual habits, new research questions can easily emerge on the interplay between brain, body, digital artifacts, and culture. Digital scholarly workflows have already opened a wealth of new possibilities for creative thinking, but also constraints, and how a possible further decline in traditional longform reading of printed books and printouts will affect the creative thinking of scholars is an urgent question. Furthermore, new technologies, such as chatbots (Bard, Bing, and ChatGPT) are constantly being developed, and they will influence many elements and relations in the cultural–cognitive systems of academia. The question is how and what the implications are, and distributed cognition perspectives could be a productive way of framing these questions. A topic that also needs more examination is the interaction between research policies, digital technologies, impact factors, and academic work, that is on the cultural situatedness of research.

Furthermore, in the “Discussion” section, it was mentioned that the interviewees were proficient users of digital tools for encoding, categorizing, and analyzing data, and more importantly of tools for mining and harvesting data. Without having pursued this topic in the interviews, the participants nevertheless gave a clear impression that most of their data was collected from digital sources, such as text corpora, online newspapers, social media, databases, online questionnaires, and e-mail interviews. If this is a widespread tendency, it raises basic questions within philosophy of science on how social scientists acquire their understanding of society and of social interaction. If digital technologies come to completely dominate research and intellectual habits, a pertinent question is how such a development will influence and color the researchers' depiction of the human experience and of the world.

## Data availability statement

The datasets presented in this article are not readily available because the interview recordings are encrypted and confidential. Requests to access the datasets should be directed to terje.hillesund@uis.no.

## Ethics statement

The studies involving human participants were reviewed and approved by Sikt - Norwegian Agency for Shared Services in Education and Research. The patients/participants provided their written informed consent to participate in this study.

## Author contributions

TH is the sole contributor to the article and corresponding author.
